# Simulation of haemodynamic flow in head and neck cancer chemotherapy

**DOI:** 10.1186/1475-925X-10-104

**Published:** 2011-12-02

**Authors:** Stephan Rhode, Manosh C Paul, Eckhard Martens, Duncan F Campbell

**Affiliations:** 1 Department of Mechanical Engineering, Karlsruhe Institute of Technology, 76131 Karlsruhe, Germany; 2 School of Engineering, University of Glasgow, Glasgow G12 8QQ, UK; 3 Department of Mechanical Engineering, University of Applied Sciences, 76133 Karlsruhe, Germany; 4 Department of Oral & Maxillofacial Surgery, Queen Margaret's Hospital, Fife, UK

**Keywords:** patient specific model, blood flow dynamics, non-Newtonian, chemotherapy, multiphase model

## Abstract

**Background:**

In recent years, intra arterial chemotherapy has become an important component in head and neck cancer treatment. However, therapy success can vary significantly and consistent treatment guidelines are missing. The purpose of this study was to create a computer simulation of the chemical agent injection in the head and neck arteries to investigate the distribution and concentration of the chemical.

**Methods:**

Realistic three dimensional patient specific geometry was created from image scan data. Pulsatile blood flow, turbulence, the chemical agent injection via a catheter, and the mixture between blood and the chemical were then simulated through the arterial network by computational fluid dynamics software.

**Results:**

The results show a consistent chemical distribution throughout all the arteries and this is ineffective. In addition, due to high wall shear stress and turbulence at the inner bifurcation wall, serious complications during the treatment could occur, for instance haemolysis or thrombosis.

**Conclusions:**

The modelled catheter position is insufficient to provide a high chemical agent concentration in the desired tumour feeding artery, which is vital for therapy success.

## 1 Background

In head and neck cancer treatment and particularly in oral cancer treatment, surgery or radiotherapy alone or radiotherapy in combination with chemotherapy is generally administered. All modalities can lead to significant problems with swallowing and speech with consequent reduction in life quality. The general aim in cancer treatment is to approach maximum tumour control with minimum related side effects. In recent years, chemotherapy has become more relevant and is now an important treatment component in combined modality approaches, several studies in this subject are summarized in [[1], p. 127]. Studies, using concurrent chemoradiation in comparison with radiation alone, are commonly randomized trials, which vary in radiation dose, fractionation schedule, and administered chemotherapy. The dosage scheme, which is usually given in mass per body surface area (mg/m^2^), as well as the administration method of the chemical is vital for the prognosis.

Using intra-arterial infusion improved the success of treatment. This is because of the arteries that supply specific tissue, detailed in [2,3]. This technique allows the supply of much higher chemical agent concentration directly to the tumour than intra-venous infusion. The latter, distributes the chemical agent throughout the whole body and causes increased side effects. Because of the patient specific artery geometry and resultant haemodynamics, there is no method that always leads to success, and this is discussed in [4]. The knowledge about the chemical agent concentration in blood and the technique by which the tumours were infused is vital for therapy success [5]. The aim is to supply a high application rate of chemical in a specified target region inside the arterial network.

Advances in computational methods and three-dimensional imaging techniques allow improved understanding of haemodynamic flow in the cardiovascular network. Computational fluid dynamics (CFD) solves differential equations of fluid flows numerically and is an excellent technique for analysing and displaying blood flow in the head and neck arterial network. In contrast to previous studies [6-9], using two or three-dimensional ideal geometry, this project deals with real geometry obtained from image-scan segmentation of a patient [10-14].

Simulation of patient specific two-component flow, using various injection positions and parameters, for instance the mass flow rate of the chemical agent, should deepen the knowledge of chemical agent distribution in the artery network, which is the aim of this study. If an accurate computer simulation is achievable in a sufficiently short time, patient specific computation would provide the basis of comparing the effect of several treatment methods and parameters prior to therapy. A two-component transient CFD model can assist medical scientists to select the most appropriate treatment strategy.

## 2 Methods

Considering the pulsatile character of blood flow, a transient CFD model is required. The model is reduced to the interesting region of the common carotid artery (CCA) bifurcation, the lingual artery (LA), facial artery (FA) and superior thyroid artery (STA) branch, including two-component flow. In particular the CFD model comprises the two liquids blood (continuous component or primary phase) and the chemical agent cisplatin (dispersed component or secondary phase). Cisplatin is injected via a catheter into the arterial blood flow. Due to this, the catheter has to be considered as thin pipe inside the CCA, because the catheter walls cause wall friction and influence the haemodynamic flow pattern.

**Figure 1:**
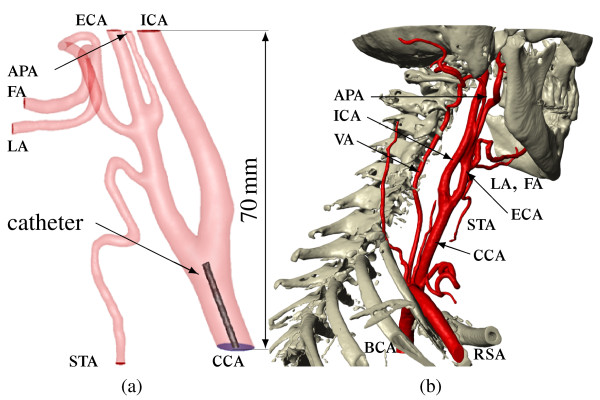
**Two-component CFD model considering the cisplatin injection through a catheter, right before the CCA bifurcation in Figure (a)**. Figure (b) shows the right arterial network and skull source volume model as result of the CT image segmentation in Scanip™. APA-ascending pharyngeal artery; BCA-brachiocephalic artery; CCA-common carotid artery; ECA-external carotid artery; FA-facial artery; ICA-internal carotid artery; LA-lingual artery; RSA-right subclavian artery; STA-superior thyroid artery; VA-vertebral artery.

A patient specific model was created with the image segmentation software Scanip™ [15] from CT-scans, presented in Figure 1. The catheter is an ideal pipe and in clinical practice has an inner diameter of 0.625 mm, an outer diameter of 0.924 mm and the common dose rate is approximately 50 ml/20 s. Four different injection positions are common:

• Short before the common carotid bifurcation.

• Internal carotid artery at the height of the superior thyroid branch.

• Lingual shunt.

• Inside the lingual artery.

It was decided to compute the first injection position, because the catheter can be modelled as straight ideal pipe, shown in Figure 1(a). Small pipe geometry creation using few voxels is complicated in Scanip™. Hence, it is impossible to model the catheter entirely including the inner wall in such small dimensions. A catheter outer wall diameter of approximately 1.2 mm (this is 30% larger than the real catheter) and tolerable wall smoothness was achieved with the CAD primitive generation tools in Scanip™. An additional volume at the top end of the catheter was created to obtain a catheter-blood interface in the following mesh import in Gambit™ [16]. Using the split face tool in Gambit™, this interface was divided by a circle of 0.6 mm diameter and an ideal circular face for the catheter outflow boundary condition was obtained. This face is slightly smaller than the real catheter inner diameter. Since the catheter inner wall is not considered in the model, it was decided to neglect the cisplatin flow inside the catheter entirely and the catheter volume remains unmeshed. Otherwise recirculation would occur inside the catheter near the catheter-blood interface. Additionally the steady ideal pipe flow of cisplatin inside the catheter is entirely solvable with analytical equations.

The mesh consists of hexahedron elements in the vessel core and tetrahedron elements adjacent to walls. A mesh independence test was done in a previous steady simulation on the highest blood flow rate of CCA using wall *y*^+ ^and pressure gradient adaption [17]. Due to just slightly monitor data changes after the mesh adaption, the initial mesh of counting 224,115 cells was taken.

**Figure 2:**
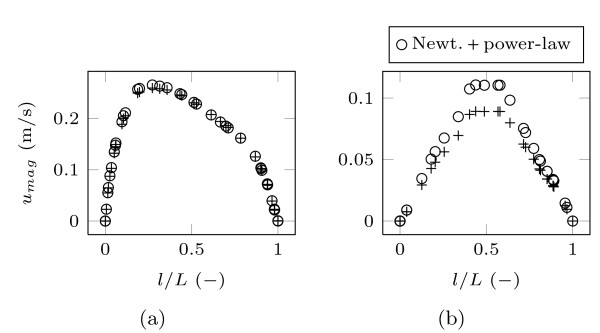
**Velocity profiles over dimensionless vessel diameter with various blood models on a low Reynolds number, taken from our previous study **[17]. Figure (a) shows the RSA with an approximately diameter of 9.3 mm, Figure (b) shows the velocity profile for the smaller FA with a vessel diameter of 2.9 mm. The non-Newtonian power-law approach results in lower velocity values, due to more viscosity on low shear rate in the FA in Figure (b). This effect was not observed in the larger RSA in Figure (a).

A prior one-phase steady cause and effect study was carried out with various Reynolds numbers and viscous models for blood [17]. In this previous study it was discovered that a non-Newtonian material model was compulsory for blood. Comparing the velocity magnitude on diametrical line probes at several outlets, significant lower velocity values were observed for the non-Newtonian models in small arteries, shown in Figure 2. In particular, a maximum offset of 20% was computed at the FA outlet between the Newtonian and the power-law non-Newtonian model due to the very small FA diameter, approximately 2.9 mm. Moreover, the shear stress transport (SST) *k*-*ω *turbulence model was found to be appropriate for the whole velocity range and of superior robustness.

**Figure 3:**
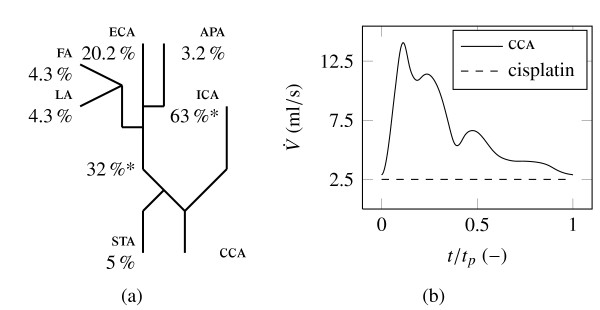
**Time continuous flow rate weighting off all outlets using the outflow boundary condition in Figure (a)**. The asterisked values are the original time averaged data of [21]. The CCA flow rate inlet condition in Figure (b) is realized as a transient table with 200 equal distant timesteps (0.0047s timestep size). The cycle time *t_p _*is 0.923s, which is equal to 65 heart beats per minute.

The boundary conditions comprise two mass flow inlets, CCA and catheter, and six outflow outlets with adjusted flow rate weighting. Several measured data dealing with CCA blood flow pattern is presented in [18-21]. It is assumed that the additional catheter inlet in the present model does not change the general flow division in the CCA bifurcation, due to the modelled catheter position still being inside the CCA. This is not advisable if the injection position moves downstream, for instance to the external carotid artery (ECA). Unfortunately it is impossible to consider a time dependent flow rate weighting of the CCA into the ECA and internal carotid artery (ICA) in the transient solver of Fluent™ as a boundary condition for each outlet. Hence, only a time averaged flow rate weighting could be used. The time averaged mass loss (ICA + ECA)/CCA is reported at roughly 5% in [21] and compared with two additional sources in Table 1. Regarding this 5% to the STA branch, it is assumed that each branch of the ICA will have the same proportion of approximately 13.5% of the ICA mass flow. The time averaged mass flow division was calculated for the ECA, STA, LA, FA and ascending pharyngeal artery (APA) outlet and assumed for the ICA outlet. Figure 3(a) provides an overview of the time continuous flow rate weighting for all outflow conditons.

**Table 1:** Measurements of the cycle averaged CCA rate of flow division in ml/min and the relative outflow/inflow mass loss

reference	$\dot{V}$ ICA	$\dot{V}$ ECA	$\dot{V}$ CCA	loss
[21]	245	125	389	4.9%

[32]	248	95	370	7.0%

[33]	255	95	376	6.9%

The most appropriate data for the CCA inlet is given in [21] obtained from older volunteers and shown with the solid line in Figure 3(b). However, it can be expected that because of the stress during chemotherapy, the heart rate should be higher than the reported 65 beats per minute in this source. Additionally, the study was made with healthy volunteers. Hence, it is not known how a catheter will affect the reported rate of flow range. These two inlet conditions are converted into a mass flux boundary in the Fluent™ simulation. In particular a transient table for the CCA and a constant value for cisplatin is used. The volume fraction of the dispersed phase cisplatin was set to 100% at the cisplatin inlet and 0% at the CCA inlet.

Blood is modelled as a non-Newtonian fluid using the Carreau model (1) with a constant density of *ρ *= 1056 kg/m^3^. The material constants for the haematocrit value of *Ht *= 45% and the temparature of *T *= 310.15 K are given in [[22], pp. 236-237]. The maximum Reynolds number of the pulsatile blood flow is 856, which indicates laminar blood flow through the whole cycle.

(1) $$\begin{array}{c} \mu \left(\dot{\gamma }\right)=\mu_{\infty }+\left(\mu_{0}-\mu_{\infty }\right){[1+{\left(\lambda_{t}\dot{\gamma }\right)}^{2}]}^{\left(n-1\right)∕2},\\ \text{where}\;\mu_{0}=0.056\;\text{Pa s},\;\mu_{\infty }=0.00345\;\text{Pa s},\\ \lambda_{t}=3.313\;\text{s},\;n=0.3568. \end{array}$$

The Newtonian material model was used for cisplatin with properties of water, *ρ *= 998.2 kg/m^3^and *μ *= 0.001003 Pa s.

The Reynolds number of the steady cisplatin flow inside the catheter is 5331. This is far above the commonly known threshold for laminar pipe flow *Re *= 2300. Turbulence is considered through the SST*k*-*ω *model. At the laminar CCA inlet *k *and *ω *is set to 0. At the turbulent cisplatin inlet on top of the catheter *k *= 0.359 m^2^/s^2 ^and *ω = *26165 s ^-1^, determined by using an approach for fully developed turbulent pipe flows (2) dealt with in [[16], ch. 7.2.2].

(2) $$\begin{array}{c} k=\frac{3}{2}{\left(u_{mag}\cdot I\right)}^{2}\;\text{with}\;I=0.16\cdot Re^{-\frac{1}{8}},\\ \omega =\frac{k^{1∕2}}{C_{\mu }^{1∕4}\cdot l_{T}}\;\text{with}\;C_{\mu }=0.09\;\text{and}\;l_{T}=0.07\cdot d_{h}. \end{array}$$

All walls are rigid and a non-slip condition is prescribed. The mixing model was used including slip velocity and implicit body force effects. Assuming a bubble size of 0.1 mm for the dispersed phase cisplatin, a hydraulic diameter of *d_h _*= 6 mm for the CCA and a maximum blood velocity of 0.8 m/s, a Stokes number of 0.021 was calculated using the equations (3), dealt with in [[23], p. 194]. This is much smaller than one and means that all cisplatin bubbles will closely follow the blood flow. A strong coupling between blood and cisplatin is supposed.

(3) $$St=\frac{\tau_{d}}{\tau_{c}}\;\text{with}\;\tau_{d}=\frac{\rho_{d}\cdot {d_{d}}^{2}}{18\mu_{c}}\;\text{and}\;\tau_{c}=\frac{d_{h}}{u_{mag}}$$

After testing one initial cycle using 100 time steps, the transient simulation proceeded in batch mode using 200 equal distant time steps for one additional cycle. Double precision, first order discretization and the pressure based segregated PISO solver including skewness and neighbour correction of one, were used with a convergence criteria of 1 · 10^-7 ^for the cisplatin volume fraction residual. All other settings remained at Fluent™ default values. Data for each 10 th time step was saved. The computing time required approximately four days, shared by three parallel processors.

## 3 Results and discussion

**Figure 4:**
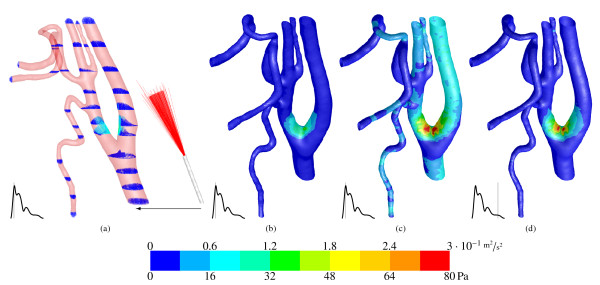
**In (a) vectors of velocity magnitude coloured by turbulent kinetic energy *k *are presented (range above the colour bar)**. The catheter on the right is displaced from its origin in the CCA centre. The mean velocity at CCA inlet is 0.38 m*s*^-1 ^and at cisplatin inlet 7.66 m*s*^-1^. Figure (b) shows the related contour plot of *k*. Figure (c) and (d) are plots of wall shear stress in systole and diastole (range below the colour bar).

Figure 4(a) shows vectors of velocity magnitude in several cross sections coloured by the turbulent kinetic energy *k *in the time step with the highest blood volume flow rate. The catheter inlet vectors are not exactly perpendicularly aligned with the catheter axis. The cisplatin flow is frontal orientated towards the inner wall of the CCA bifurcation with a small incline towards the ECA. Creation of the catheter geometry in CAD software would be useful to obtain a proper plain catheter front surface. This would be perpendicular, ideally cylindric and the catheter wall would be smooth. Because of the turbulent Reynolds number inside the catheter, turbulence is introduced in the blood flow through the cisplatin inlet. And it occurs at both inner walls after the CCA bifurcation in the ICA and ECA. Sutera and Mehrjardi [24] reported in their study that the red blood cells deform gradually toward a smooth ellipsoidal shape by the alternation of the magnitude in the shear stress from 10 Pa to 250 Pa. The region of fluctuating stresses associated with high turbulent kinetic energy, that indicates high turbulence and is shown in Figure 4(b), is therefore critical in matters of red blood cell damage (haemolysis), thrombosis (blood clotting) or even a stroke. Additionally, the CCA bifurcation is known as region with a high possibility of arteriosclerosis for elderly patients. The turbulent cisplatin flow could have an abrasive effect and release fatty particles.

Furthermore, in consequence of the frontal orientated cisplatin inlet stream towards the CCA bifurcation wall, the wall shear stress shows a peak (≈ 80 Pa) in this region, presented in Figure 4(c) and 4(d) for systole and diastole, respectively. This level of high wall shear stress can potentially cause acute vascular endothelial changes as it was found in [25] that the acute yield stress is approximately 37.9 Pa.

Due to the toxic properties of cisplatin, this catheter outflow position can cause several complications, such as local inflammation and neurological complications, for instance headache and facial paresis, [3].

**Figure 5:**
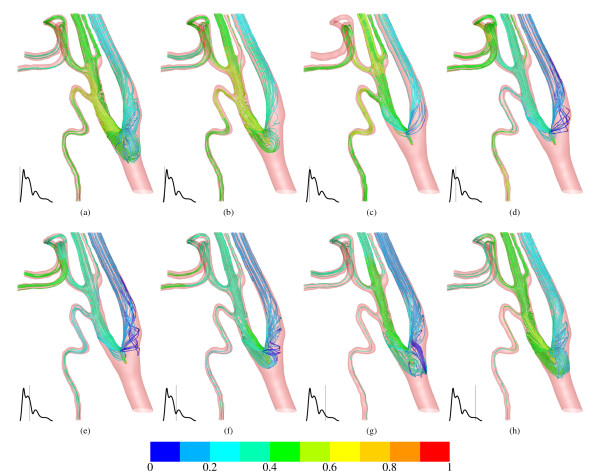
**Path lines with origin cisplatin inlet coloured by volume fraction of cisplatin during one cardiac cycle**. The catheter is hidden. The blood flow condition at the CCA inlet is shown in each picture on the bottom left.

The cisplatin distribution during one cardiac cycle can be described in detail by using Figure 5. In general, the chemical agent cisplatin distributes in all arteries, as to be expected for this catheter position. This is undesirable, because only a high cisplatin concentration in a specific region gives maximum tumour control with minimum related side effects. Prior systole, the path lines indicate recirculation upstream in the CCA. The cisplatin volume fraction in the ECA is larger than in the ICA. This is explained by the flow rate weighting conditions of Figure 3(a). With the start of systole and increasing blood flow, the cisplatin concentration decreases in the larger arteries ECA and ICA, Figure 5 (c)-(e), and the recirculation region disappears. There is a time shift in cisplatin volume fraction reduction to note between ECA and LA with FA. This is shown in Figure 5(d). This shift is retained until the beginning of the diastole in Figure 5(f) when the volume fraction in ECA, LA and FA are approximately equal. At the end of diastole in Figure 5(g) a similar condition is observed as in Figure 5(a). This suggests a periodic cisplatin distribution characteristic controlled by the cardiac cycle. It also shows the importance of considering a pulsatile blood flow boundary condition. If the simulation was steady, a delay in cisplatin concentration at various artery limits would be invisible in the simulation results.

**Figure 6:**
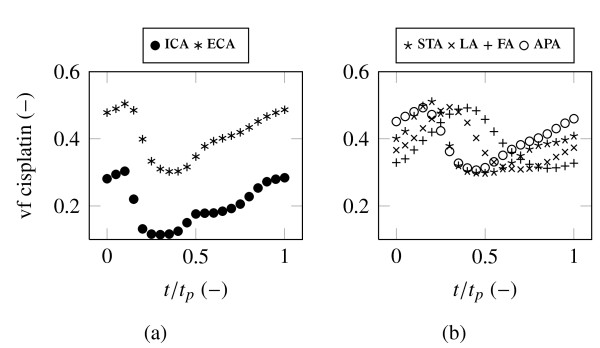
**Mass weighted average cisplatin volume fraction at the outlets over one cardiac cycle**.

The mass weighted average cisplatin volume fraction for all 21 saved time steps at all model outlets is presented in Figure 6. The discussed delay of cisplatin concentration between ICA and LA with FA is good to observe. In particular, the functions of ICA, ECA, STA and APA show a qualitatively similar shape contrary to the functions of LA and FA. Apart from the ICA all arteries have an approximately equal cisplatin concentration range between 0.3 and 0.5. It is not expected that a variation in cisplatin dosage will change this uniform distribution, rather a catheter position further downstream could have a positive effect in this matter.

**Figure 7:**
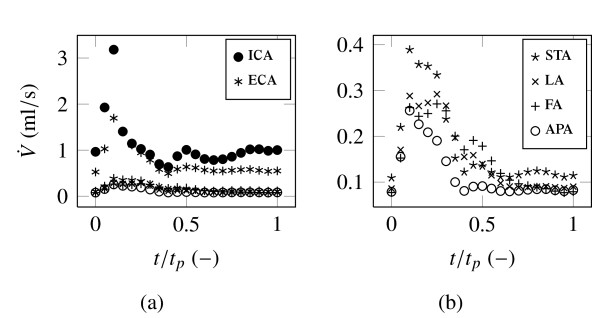
**Cisplatin rate of flow at the outlets**. Figure (b) shows the outlets STA, LA, FA and APA of Figure (a) separately with a finer ordinate resolution.

Additionally, the time dependent cisplatin concentration functions, observed for all outlets, indicate that the time continuous boundary condition at the cisplatin inlet is not the most efficient technique for applying high chemical agent dosages. It is possible that an unsteady injection at a particular beneficial time range on higher dosage rate could enhance the cisplatin concentration and reduce side effects, such as local inflammation at artery walls, during the remaining period. An advantageous time range for injection would be between *t*/*t_p _*= 0-0.3 during peak systole. Furthermore, it is possible that recirculation and the exposure time on high turbulence could be reduced by time dependent injection during systole. Future simulations are required to provide evidence for this assumptions. In absolute values, Figure 7 represents the rate of flow of the chemical. The prescribed flow rate weighting is the reason for the low level of STA, LA, FA and APA compared with ECA and ICA.

## 4 Conclusions

The distribution and concentration of the chemical agent cisplatin have been investigated in a realistic three dimensional patient specific geometry of human head and neck arteries. The used injection position gives an ineffective cisplatin division. This means that the chemical agent is distributed in all arteries and not in one specific tumour feeding artery, a fact of great importance. High wall shear stress and turbulence near the CCA bifurcation inner walls can cause serious harm, for instance acute vascular endothelial changes or haemolysis. It should be expected that an injection position in the core of the artery further downstream would reduce this high exposure. The time continuous cisplatin inlet condition seems to be ineffective during diastole, when upstream recirculation in the CCA mainly occurs. It should be investigated if a time dependent injection during the advantageous systole would lead to an equal cycle averaged cisplatin concentration with reduced side effects caused by the catheter inflow.

In blood flow CFD simulation using multiphase models, neither mass flow nor velocity outlets are practicable, due to required outlet data for each phase. This data is unknown a priori for the secondary phase cisplatin and is the aim of this simulation. An assumed flow rate weighting was used in this model based on measured data for the two main outlets ICA and ECA. It is assumed that the general blood flow division remains unchanged in comparison with measured data of healthy volunteers in the normal state. An injection position further downstream in a side branch of the ECA would probably change the blood flow division. An alternative proper methodology would be to use the pressure inlet and outlet boundary conditions, that require only the volume fraction of the secondary phase at the inlets, and adjust the pressure to a correct level. However, time dependent pressure functions for each model boundary were not available and are difficult to assess for small arteries, such as the LA and FA. The implementation of an arterial one dimensional model seams to be the superior way for future CFD simulations dealt with in [26-31].

## List of abbreviations used

APA: ascending pharyngeal artery; BCA: brachiocephalic artery; CAD: computer aided design; CCA: common carotid artery; CFD: computational fluid dynamics; CT: computer tomography; ECA: external carotid artery; FA: facial artery; ICA: internal carotid artery; LA: lingual artery; PISO: pressure implicit with splitting of operators; RSA: right subclavian artery; SST: shear stress transport; STA: superior thyroid artery; VA: vertebral artery

## Competing interests

The authors declare that they have no competing interests.

## Authors' contributions

SR, MCP and EM carried out the CFD studies while DFC provided CT images and clinical inputs. All the authors contributed to the preparation of the manuscript and then approved its final version.
